# Genetic variation at *CYP3A* is associated with age at menarche and breast cancer risk: a case-control study

**DOI:** 10.1186/bcr3662

**Published:** 2014-05-26

**Authors:** Nichola Johnson, Frank Dudbridge, Nick Orr, Lorna Gibson, Michael E Jones, Minouk J Schoemaker, Elizabeth J Folkerd, Ben P Haynes, John L Hopper, Melissa C Southey, Gillian S Dite, Carmel Apicella, Marjanka K Schmidt, Annegien Broeks, Laura J Van’t Veer, Femke Atsma, Kenneth Muir, Artitaya Lophatananon, Peter A Fasching, Matthias W Beckmann, Arif B Ekici, Stefan P Renner, Elinor Sawyer, Ian Tomlinson, Michael Kerin, Nicola Miller, Barbara Burwinkel, Frederik Marme, Andreas Schneeweiss, Christof Sohn, Pascal Guénel, Therese Truong, Emilie Cordina, Florence Menegaux, Stig E Bojesen, Børge G Nordestgaard, Henrik Flyger, Roger Milne, M Pilar Zamora, Jose Ignacio Arias Perez, Javier Benitez, Leslie Bernstein, Hoda Anton-Culver, Argyrios Ziogas, Christina Clarke Dur, Hermann Brenner, Heiko Müller, Volker Arndt, Aida Karina Dieffenbach, Alfons Meindl, Joerg Heil, Claus R Bartram, Rita K Schmutzler, Hiltrud Brauch, Christina Justenhoven, Yon-Dschun Ko, Heli Nevanlinna, Taru A Muranen, Kristiina Aittomäki, Carl Blomqvist, Keitaro Matsuo, Thilo Dörk, Natalia V Bogdanova, Natalia N Antonenkova, Annika Lindblom, Arto Mannermaa, Vesa Kataja, Veli-Matti Kosma, Jaana M Hartikainen, Georgia Chenevix-Trench, Jonathan Beesley, Anna H Wu, David Van den Berg, Chiu-Chen Tseng, Diether Lambrechts, Dominiek Smeets, Patrick Neven, Hans Wildiers, Jenny Chang-Claude, Anja Rudolph, Stefan Nickels, Dieter Flesch-Janys, Paolo Radice, Paolo Peterlongo, Bernardo Bonanni, Valeria Pensotti, Fergus J Couch, Janet E Olson, Xianshu Wang, Zachary Fredericksen, Vernon S Pankratz, Graham G Giles, Gianluca Severi, Laura Baglietto, Chris Haiman, Jacques Simard, Mark S Goldberg, France Labrèche, Martine Dumont, Penny Soucy, Soo Teo, Cheng Har Yip, Sze Yee Phuah, Belinda K Cornes, Vessela N Kristensen, Grethe Grenaker Alnæs, Anne-Lise Børresen-Dale, Wei Zheng, Robert Winqvist, Katri Pylkäs, Arja Jukkola-Vuorinen, Mervi Grip, Irene L Andrulis, Julia A Knight, Gord Glendon, Anna Marie Mulligan, Peter Devillee, Jonine Figueroa, Stephen J Chanock, Jolanta Lissowska, Mark E Sherman, Per Hall, Nils Schoof, Maartje Hooning, Antoinette Hollestelle, Rogier A Oldenburg, Madeleine Tilanus-Linthorst, Jianjun Liu, Angie Cox, Ian W Brock, Malcolm WR Reed, Simon S Cross, William Blot, Lisa B Signorello, Paul DP Pharoah, Alison M Dunning, Mitul Shah, Daehee Kang, Dong-Young Noh, Sue K Park, Ji-Yeob Choi, Mikael Hartman, Hui Miao, Wei Yen Lim, Anthony Tang, Ute Hamann, Asta Försti, Thomas Rüdiger, Hans Ulrich Ulmer, Anna Jakubowska, Jan Lubinski, Katarzyna Jaworska-Bieniek, Katarzyna Durda, Suleeporn Sangrajrang, Valerie Gaborieau, Paul Brennan, James McKay, Susan Slager, Amanda E Toland, Celine Vachon, Drakoulis Yannoukakos, Chen-Yang Shen, Jyh-Cherng Yu, Chiun-Sheng Huang, Ming-Feng Hou, Anna González-Neira, Daniel C Tessier, Daniel Vincent, Francois Bacot, Craig Luccarini, Joe Dennis, Kyriaki Michailidou, Manjeet K Bolla, Jean Wang, Douglas F Easton, Montserrat García-Closas, Mitch Dowsett, Alan Ashworth, Anthony J Swerdlow, Julian Peto, Isabel dos Santos Silva, Olivia Fletcher

**Affiliations:** 1 Breakthrough Breast Cancer Research Centre, The Institute of Cancer Research, 237 Fulham Road, London SW3 6JB, UK; 2 Division of Breast Cancer Research, The Institute of Cancer Research, 237 Fulham Road, London SW3 6JB, UK; 3 Non-communicable Disease Epidemiology Department, London School of Hygiene and Tropical Medicine, Keppel Street, London WC1E 7HT, UK; 4 Division of Genetics and Epidemiology, The Institute of Cancer Research, 15 Cotswold Road, Belmont, Sutton, Surrey SM2 5NG, UK; 5 The Academic Department of Biochemistry, The Royal Marsden Hospital, Fulham Road, London SW3 6JJ, UK; 6 Centre for Molecular, Environmental, Genetic and Analytic Epidemiology, University of Melbourne, 1-100 Gratton Street, Parkville, Melbourne, Victoria 3010, Australia; 7 Genetic Epidemiology Department, Department of Pathology, The University of Melbourne, 1-100 Gratton Street, Parkville, Melbourne, Victoria 3010, Australia; 8 Division of Molecular Pathology, Netherlands Cancer Institute, Antoni van Leeuwenhoek Hospital, Plesmanlaan 121, 1066CX Amsterdam, The Netherlands; 9 Sanquin, Radboud Universiteit Nijmegen, 6525 GA, Nijmegen, The Netherlands; 10 Warwick Medical School, University of Warwick, Coventry CV4 7AJ, UK; 11 University Breast Center, Department of Gynecology and Obstetrics, University Hospital Erlangen, Postfach 2306, D-91012 Erlangen, Germany; 12 David Geffen School of Medicine, Department of Medicine, Division of Hematology and Oncology, University of California, 10833 Le Conte Avenue, Los Angeles, CA 90095, USA; 13 Institute of Human Genetics, Friedrich Alexander University Erlangen- Nuremberg, Schlossplatz 4, 91054 Erlangen, Germany; 14 Division of Cancer Studies, NIHR Comprehensive Biomedical Research Centre, Guy’s & St. Thomas’ NHS Foundation Trust in partnership with King’s College London, Guy’s Hospital, Great Maze Pond, London SE1 9RT, UK; 15 Welcome Trust Centre for Human Genetics, University of Oxford, Roosevelt Drive, Oxford OX3 7BN, UK; 16 Oxford Biomedical Research Centre, University of Oxford, The Churchill Hospital, Old Road, Headington OX3 7LE Oxford, UK; 17 Surgery, Clinical Science Institute, Galway University Hospital and National University of Ireland, University Road, Galway, Ireland; 18 Department of Obstetrics and Gynecology, University of Heidelberg, Vosstrasse 9, 69115 Heidelberg, Germany; 19 Unit Molecular Epidemiology C080, German Cancer Research Center, DKFZ, Im Neuenheimer Feld 280, 69120 Heidelberg, Germany; 20 National Center for Tumor Diseases, University of Heidelberg, Im Neuenheimer Feld 400, 69120 Heidelberg, Germany; 21 Inserm (National Institute of Health and Medical Research), CESP (Center for Research in Epidemiology and Population Health), U1018, Environmental Epidemiology of Cancer, 101 rue de Tolbiac, Villejuif, 75654 Paris, France; 22 University Paris-Sud, UMRS 1018, 101 rue de Tolbiac, Villejuif, 75654 Paris, France; 23 Copenhagen General Population Study, Herlev Hospital, Copenhagen University Hospital, Herlev Rinvej 75, 2730 Herlev, Copenhagen, Denmark; 24 Department of Clinical Biochemistry, Herlev Hospital, Copenhagen University Hospital, Herlev Rinvej 75, 2730 Herlev, Copenhagen, Denmark; 25 Department of Breast Surgery, Herlev Hospital, Copenhagen University Hospital, Herlev Rinvej 75, Herlev, 2730 Copenhagen, Denmark; 26 Genetic and Molecular Epidemiology Group, Human Cancer Genetics Program, Spanish National Cancer Research Centre (CNIO), Calle de Melchor Fernandez Almagro, 3, 28029 Madrid, Spain; 27 Servicio de Oncología Médica, Hospital Universitario La Paz, Paseo de la Castellana, 261, 28046 Madrid, Spain; 28 Servicio de Cirugía General y Especialidades, Hospital Monte Naranco, Avda. Dres. Fernández Vega, 107 Oviedo, Spain; 29 Human Genotyping-CEGEN Unit, Human Cancer Genetics Program, Spanish National Cancer Research Centre (CNIO), Calle de Melchor Fernandez Almagro, 3, 28029 Madrid, Spain; 30 Centro de Investigación en Red de Enfermedades Raras (CIBERER), Calle de Melchor Fernandez Almagro, 3, 28029 Madrid, Spain; 31 Division of Cancer Etiology, Department of Population Sciences, Beckman Research Institute of the City of Hope, Duarte, CA, USA; 32 Department of Epidemiology, School of Medicine, 224 Irvine Hall, University of California Irvine, Irvine, California 92697-7550, USA; 33 Cancer Prevention Institute of California, 2201 Walnut Avenue, Suite 300, Fremont, California 95438, USA; 34 Division of Clinical Epidemiology and Aging Research, German Cancer Research Center, Im Neuenheimer Feld 280, 69121 Heidelberg, Germany; 35 German Cancer Consortium (DKTK), Im Neuenheimer Feld 280, 69121 Heidelberg, Germany; 36 Clinic of Gynecology and Obstetrics, Division of Tumor Genetics, Klinikum rechts der Isar, Technical University Munich, Ismaninger Strasse 22, D-81675 Munich, Germany; 37 Institute of Human Genetics, University of Heidelberg, Im Neuenheimer Feld 366, 69121 Heidelberg, Germany; 38 Division of Molecular Gyneco-Oncology, Department of Gynaecology and Obstetrics, Center of Molecular Medicine Cologne (CMMC), University Hospital of Cologne, ZMMK-Forschungsgebäude, Robert-Koch-Strasse 21, 50931 Cologne, Germany; 39 Dr. Margarete Fischer-Bosch-Institute of Clinical Pharmacology, Robert Bosch Stiftung GmbH, Heidehofstrasse 31, 70184 Stuttgart, Germany; 40 University of Tübingen, Geschwister-Scholl-Platz, 72074 Tübingen, Germany; 41 Department of Internal Medicine, Evangelische Kliniken Bonn GGmbH, Johanniter Krankenhaus, 53113 Bonn, Germany; 42 Department of Obstetrics and Gynecology, Helsinki University Central Hospital, University of Helsinki, Haartmaninkatu 2, P.O. Box 140, FIN-00029 Helsinki, Finland; 43 Department of Clinical Genetics, Helsinki University Central Hospital, Haartmaninkatu 2, P.O. Box 140, FIN-00029 Helsinki, Finland; 44 Department of Oncology, Helsinki University Central Hospital, Haartmaninkatu 2, P.O. Box 140, FIN-00029 Helsinki, Finland; 45 Division of Epidemiology and Prevention, Aichi Cancer Center Research Institute, 1-1Kanokoden, Chikusa-ku, Nagoya 464-8681, Japan; 46 Department of Obstetrics and Gynaecology, Hannover Medical School, Carl-Neuberg-Str. 1, 30625 Hannover, Germany; 47 Department of Radiation Oncology, Hannover Medical School, Carl-Neuberg-Str. 1, 30625 Hannover, Germany; 48 N.N. Alexandrov Research Institute of Oncology and Medical Radiology, 223040, p. Lesnoy, Minsk, Belarus; 49 Department of Molecular Medicine and Surgery, Karolinska Institutet, Solnavägen 1, 171 77 Solna, Stockholm, Sweden; 50 School of Medicine, Institute of Clinical Medicine, Pathology and Forensic Medicine, University of Eastern Finland, Yliopistonranta 1, P.O. Box 1627 FI-70211 Kuopio, Finland; 51 Biocenter Kuopio, Cancer Center of Eastern Finland, University of Eastern Finland, Yliopistonranta 1, P.O. Box 1627 FI-70211 Kuopio, Finland; 52 Imaging Center, Department of Clinical Pathology, Kuopio University Hospital, P.O. Box 100 FI-70029 Kuopio, Finland; 53 Imaging Center, Department of Clinical Pathology, Kuopio University Hospital, P.O. Box 100 FI-70029 Kuopio, Finland; 54 Department of Genetics, Queensland Institute of Medical Research, 300 Herston Rd, Herston, Brisbane Queensland 4006, Australia; 55 Department of Preventive Medicine, Keck School of Medicine, University of Southern California, 1975 Zonal Ave, Los Angeles, CA 90033, USA; 56 Laboratory for Translational Genetics, Department of Oncology, University of Leuven, Oude Markt 13 - bus 5005, 3000 Leuven, Belgium; 57 Vesalius Research Center, VIB, Herestraat 49, box 912, Onderwijs & Navorsing 4, Building 404-24, 3000 Leuven, Belgium; 58 Multidisciplinary Breast Center, University Hospital Gasthuisberg, Herestraat 49, 3000 Leuven, Belgium; 59 Division of Cancer Epidemiology, German Cancer Research Center (DKFZ), Im Neuenheimer Feld 280, 69120 Heidelberg, Germany; 60 Department of Cancer Epidemiology/Clinical Cancer Registry, University Clinic Hamburg-Eppendorf, Martinistrasse 52, D - 20246 Hamburg, Germany; 61 Institute for Medical Biometrics and Epidemiology, University Clinic Hamburg-Eppendorf, Martinistrasse 52, D - 20246 Hamburg, Germany; 62 Unit of Molecular Bases of Genetic Risk and Genetic Testing, Department of Preventive and Predictive Medicine, Fondazione IRCCS Istituto Nazionale dei Tumori (INT), Via Venezian 1, 20133 Milan, Italy; 63 IFOM, Fondazione Istituto FIRC di Oncologia Molecolare, Via Adamello 16, 20139 Milan, Italy; 64 Division of Cancer Prevention and Genetics, Istituto Europeo di Oncologia (IEO), Via Giuseppe Ripamonti 435, 20141 Milan, Italy; 65 Cogentech Cancer Genetic Test Laboratory, IFOM-IEO Campus, Via Adamello16, 20139 Milan, Italy; 66 Department of Laboratory Medicine and Pathology, Division of Experimental Pathology, Mayo Clinic, 200 First Street SW, Rochester, MN 55905, USA; 67 Department of Health Sciences Research, Mayo Clinic, 200 First Street SW, Rochester, MN 55905, USA; 68 Cancer Epidemiology Centre, The Cancer Council Victoria, 615 St Kilda Road, Melbourne, Victoria 3004, Australia; 69 Department of Medicine, McGill University and Division of Clinical Epidemiology, McGill University Health Centre, Royal Victoria Hospital, 687 Pine Avenue West, Montréal, Québec H3A 1A1, Canada; 70 Department of Social and Preventive Medicine and Department of Environmental and Occupational Health at Work, University of Montréal, Marguerite d'Youville Pavilion, 2375 Côte Ste-Catherine, Suite 4095, Montréal, Québec H3T 1A8, Canada; 71 Cancer Genomics Laboratory, Centre Hospitalier Universitaire de Québec Research Center and Laval University, 2325 Rue de l'Université, Québec City, Québec G1V 0A6, Canada; 72 Breast Cancer Research Unit, University of Malaya Cancer Research Institute, Faculty of Medicine, University of Malaya, 50603 Kuala Lumpur, Malaysia; 73 Cancer Research Initiatives Foundation, Sime Darby Medical Centre Subang Jaya, 1, Jalan SS 12 / 1A, 47500 Subang Jaya, Selangor Darul Ehsan, Malaysia; 74 Singapore Eye Research Institute, National University of Singapore, Singapore National Eye Centre, 11 Third Hospital Avenue, Singapore 168751, Singapore; 75 Department of Genetics, Institute for Cancer Research, Oslo University Hospital, The Norwegian Radium Hospital, N-0310 Oslo, Norway; 76 Faculty of Medicine (Faculty Division Ahus), University of Oslo, Sogn Arena, Klaus Torgårds vei 3, 2. etg, 0372 Oslo, Norway; 77 Division of Epidemiology, Department of Medicine, Vanderbilt Epidemiology Center, Vanderbilt-Ingram Cancer Center, Vanderbilt University School of Medicine, 1161 21st Ave S # T1217, Nashville, TN 37232, USA; 78 Laboratory of Cancer Genetics and Tumor Biology, Department of Clinical Chemistry and Biocenter Oulu, University of Oulu, Oulu University Hospital, Kajaanintie 50, 90220 Oulu, Finland; 79 Department of Oncology, Oulu University Hospital, University of Oulu, Kajaanintie 50, 90220 Oulu, Finland; 80 Department of Surgery, Oulu University Hospital, University of Oulu, Kajaanintie 50, 90220 Oulu, Finland; 81 Samuel Lunenfeld Research Institute, Mount Sinai Hospital, 982 - 600 University Avenue, Toronto, Ontario M5G 1X5, Canada; 82 Department of Molecular Genetics, University of Toronto, Medical Science Building, Room 4386, 1 King's College Cir, Toronto, Ontario M5S 1A8, Canada; 83 Division of Epidemiology, Dalla Lana School of Public Health, University of Toronto, 6th floor, 155 College St, Toronto, Ontario M5T 3M7, Canada; 84 Ontario Cancer Genetics Network, 620 University Avenue, Toronto, Ontario M5G 2L7, Canada; 85 Department of Laboratory Medicine and Pathobiology, University of Toronto, Medical Sciences Building, 6th Floor, 1 King's College Cir, Toronto, Ontario M5S 1A8, Canada; 86 University Health Network, R. Fraser Elliott Building, 1st Floor, 190 Elizabeth St., Toronto, Ontario M5G 2C4, Canada; 87 Department of Human Genetics & Department of Pathology, Leiden University Medical Center, Einthovenweg 20, 2333 ZC, Leiden, The Netherlands; 88 Division of Cancer Epidemiology and Genetics, National Cancer Institute, 9609 Medical Center Drive, Rockville, MD 20850, USA; 89 Department of Cancer Epidemiology and Prevention, M. Sklodowska-Curie Memorial Cancer Center & Institute of Oncology, Roentena 5, 02-781 Warsaw, Poland; 90 Department of Medical Epidemiology and Biostatistics, Karolinska Institutet, Solnavägen 1, Stockholm 17177, Sweden; 91 Department of Medical Oncology, Family Cancer Clinic, Erasmus University Medical Center, Groene Hilledijk 301, Rotterdam, EA, 3075, The Netherlands; 92 Department of Medical Oncology, Josephine Nefkens Institute, Erasmus University Medical Center, Groene Hilledijk 301, 3075 EA, Rotterdam, The Netherlands; 93 Department of Clinical Genetics, Family Cancer Clinic, Erasmus University Medical Center, Groene Hilledijk 301, 3075 EA, Rotterdam, The Netherlands; 94 Human Genetics Division, Genome Institute of Singapore, 60 Biopolis St, Singapore 138672, Singapore; 95 Institute for Cancer Studies, Department of Oncology, CRUK/YCR Sheffield Cancer Research Centre, University of Sheffield, 385a Glossop Road, Sheffield S10 2HQ, UK; 96 Academic Unit of Surgical Oncology, Department of Oncology, CRUK/YCR Sheffield Cancer Research Centre, University of Sheffield, 385a Glossop Road, Sheffield S10 2HQ, UK; 97 Academic Unit of Pathology, Department of Neuroscience, University of Sheffield, 385a Glossop Road, Sheffield S10 2HQ, UK; 98 International Epidemiology Institute, 1455 Research Blvd, Rockville, MD 20850, USA; 99 Department of Epidemiology, Harvard School of Public Health, 677 Huntington Avenue, Boston, MA 02115, USA; 100 Channing Division of Network Medicine, Harvard Medical School, 181 Longwood Avenue, Boston, MA 02115, USA; 101 Dana-Farber/Harvard Cancer Center, 450 Brookline Ave, Boston, MA 02215, USA; 102 Department of Oncology, Centre for Cancer Genetic Epidemiology, University of Cambridge, Strangeways Research Laboratory, Worts Causeway, Cambridge CB1 8RN, UK; 103 Seoul National University College of Medicine, Yongeon-103 Daehangno, Jongno-gu, Seoul 110-799, Korea; 104 Department of Preventive Medicine, Seoul National University College of Medicine, Yongeon-103 Daehangno, Jongno-gu, Seoul 110-799, Korea; 105 Department of Biomedical Science, Seoul National University Graduate School, Yongeon-103 Daehangno, Jongno-gu, Seoul 110-799, Korea; 106 Cancer Research Institute, Seoul National University, Yongeon-103 Daehangno, Jongno-gu, Seoul 110-799, Korea; 107 Department of Surgery, Yong Loo Lin School of Medicine, National University of Singapore, 1E, Kent Ridge Road, Singapore 119228, Singapore; 108 National University Health System, 1E, Kent Ridge Road, Singapore 119228, Singapore; 109 Saw Swee Hock School of Public Health, National University of Singapore, MD3, 16 Medical Drive, Singapore 117597, Singapore; 110 Division of General Surgery, National University Health System, 1E, Kent Ridge Road, Singapore 119228, Singapore; 111 Molecular Genetics of Breast Cancer, German Cancer Research Center (DKFZ), Im Neuenheimer Feld 280, 69120 Heidelberg, Germany; 112 Division of Molecular Genetic Epidemiology, German Cancer Research Center (DKFZ), Im Neuenheimer Feld 280, 69120 Heidelberg, Germany; 113 Center for Primary Health Care Research, University of Lund, Paradisgatan 5, SE-221 00 Lund, Malmö, Sweden; 114 Institute of Pathology, Städtisches Klinikum Karlsruhe, Moltkestrasse 90, 76133 Karlsruhe, Germany; 115 Frauenklinik der Stadtklinik Baden-Baden, Balger Strasse 50, 76532 Baden-Württemberg, Germany; 116 Department of Genetics and Pathology, Pomeranian Medical University, Rybacka 1, 70-204 Szczecin, Poland; 117 Postgraduate School of Molecular Medicine, Warsaw Medical University, Żwirki i Wigury 61, 02-091 Warsaw, Poland; 118 National Cancer Institute, 268/1 Rama VI Road, Rajathevi, Bangkok 10400, Thailand; 119 International Agency for Research on Cancer, 150 Cours Albert Thomas, 69372 Lyon, CEDEX 08, France; 120 Department of Molecular Virology, Immunology and Medical Genetics, Comprehensive Cancer Center, The Ohio State University, 410 W. 10th Avenue, Columbus, OH 43210, USA; 121 Molecular Diagnostics Laboratory, IRRP, National Centre for Scientific Research “Demokritos”, Aghia Paraskevi Attikis 153 10, Athens, Greece; 122 College of Public Health, China Medical University, No.91, Hsueh-Shih Road, Taichung 40402, Taiwan; 123 Institute of Biomedical Sciences, Academia Sinica, 2 Academia Road, Nankang, Taipei 115, Taiwan; 124 Department of Surgery, Tri-Service General Hospital, No.325, Sec.2 Chenggong Road, Taipei City 114, Neihu District, Taiwan; 125 Department of Surgery, National Taiwan University Hospital, No.1, Changde Street, Taipei City 10048, Zhongzheng District, Taiwan; 126 Cancer Center, Kaohsiung Medical University Chung-Ho Memorial Hospital, No.100, Tzyou 1st Road, Kaohsiung 807, Taiwan; 127 Department of Surgery, Kaohsiung Medical University Chung-Ho Memorial Hospital, No.100, Tzyou 1st Road, Kaohsiung 807, Taiwan; 128 McGill University and Génome Québec Innovation Centre, 740, Dr. Penfield Avenue, Room 7104, Montréal, Québec H3A 0G1, Canada; 129 Department of Public Health and Primary Care, Centre for Cancer Genetic Epidemiology, University of Cambridge, Strangeways Research Laboratory, Worts Causeway, Cambridge CB1 8RN, UK

## Abstract

**Introduction:**

We have previously shown that a tag single nucleotide polymorphism (rs10235235), which maps to the *CYP3A* locus (7q22.1), was associated with a reduction in premenopausal urinary estrone glucuronide levels and a modest reduction in risk of breast cancer in women age ≤50 years.

**Methods:**

We further investigated the association of rs10235235 with breast cancer risk in a large case control study of 47,346 cases and 47,570 controls from 52 studies participating in the Breast Cancer Association Consortium. Genotyping of rs10235235 was conducted using a custom Illumina Infinium array. Stratified analyses were conducted to determine whether this association was modified by age at diagnosis, ethnicity, age at menarche or tumor characteristics.

**Results:**

We confirmed the association of rs10235235 with breast cancer risk for women of European ancestry but found no evidence that this association differed with age at diagnosis. Heterozygote and homozygote odds ratios (ORs) were OR = 0.98 (95% CI 0.94, 1.01; *P* = 0.2) and OR = 0.80 (95% CI 0.69, 0.93; *P* = 0.004), respectively (*P*_trend_ = 0.02). There was no evidence of effect modification by tumor characteristics. rs10235235 was, however, associated with age at menarche in controls (*P*_trend_ = 0.005) but not cases (*P*_trend_ = 0.97). Consequently the association between rs10235235 and breast cancer risk differed according to age at menarche (*P*_het_ = 0.02); the rare allele of rs10235235 was associated with a reduction in breast cancer risk for women who had their menarche age ≥15 years (OR_het_ = 0.84, 95% CI 0.75, 0.94; OR_hom_ = 0.81, 95% CI 0.51, 1.30; *P*_trend_ = 0.002) but not for those who had their menarche age ≤11 years (OR_het_ = 1.06, 95% CI 0.95, 1.19, OR_hom_ = 1.07, 95% CI 0.67, 1.72; *P*_trend_ = 0.29).

**Conclusions:**

To our knowledge rs10235235 is the first single nucleotide polymorphism to be associated with both breast cancer risk and age at menarche consistent with the well-documented association between later age at menarche and a reduction in breast cancer risk. These associations are likely mediated via an effect on circulating hormone levels.

## Introduction

Family history is a well-established risk factor for breast cancer. First-degree relatives of women with breast cancer have an approximately twofold increased risk of developing the disease relative to the general population [1]. Twin studies are consistent with this familial clustering having, at least in part, a genetic origin [2,3]. Mutations in high-risk susceptibility genes (mainly *BRCA1* and *BRCA2*) explain most large multiple-case families, but account for only 15 to 20% of the excess familial risk [4]. Genome-wide association studies [5,6] have identified more than 70 common variants that are associated with breast cancer susceptibility but they account for only another approximately 15% of the excess familial risk. The so-called ‘missing heritability’ may be explained by common variants with very small effects and/or by rarer variants with larger effects, neither of which can be identified by current genome-wide association studies. A statistically efficient alternative is to increase power by trying to identify variants associated with known quantitative phenotypic markers of susceptibility to breast cancer [7], and then to test them for association with breast cancer risk. This approach might also improve our understanding of the biological mechanisms involved in breast cancer pathogenesis.

Endogenous sex hormones are well-established risk factors for breast cancer in postmenopausal women [8]; the evidence in premenopausal women is less consistent, with some, but not all, studies suggesting an association between higher circulating levels of estrogens and increased breast cancer risk [9-17]. Genetic factors influence the levels of endogenous sex hormones [18] and therefore single nucleotide polymorphisms (SNPs) in genes regulating these hormonal pathways are good candidates for being breast cancer predisposition variants. We have previously studied 642 SNPs tagging 42 genes that might influence sex hormone levels in 729 healthy premenopausal women of European ancestry in relation to cyclic variations in oestrogen levels during the menstrual cycle. We found that the minor allele of rs10273424, which maps 50 kb 3′ to *CYP3A5*, was associated with a reduction of 22% (95% confidence interval (CI) = –28%, –15%; *P* = 10^-9^) in levels of urinary oestrone glucuronide, a metabolite that is highly correlated with serum oestradiol levels [19]. Analysis of 10,551 breast cancer cases and 17,535 controls of European ancestry demonstrated that the minor allele of rs10235235, a proxy for rs10273424 (*r*^*2*^ = 1.0), was also associated with a weak reduction in breast cancer risk but only in women aged 50 years or younger at diagnosis (odds ratio (OR) = 0.91, 95% CI = 0.83, 0.99; *P* = 0.03) [19].

The aim of the present study was to further investigate an association between rs10235235 and breast cancer risk using a much larger set of subjects – the Breast Cancer Association Consortium (BCAC) – comprising data from 49 additional studies, and to assess whether there was evidence of effect modification by age at diagnosis, ethnicity, age at menarche or tumour characteristics.

## Materials and methods

### Sample selection

Samples for the case–control analyses were drawn from 52 studies participating in the BCAC: 41 studies from populations of predominantly European ancestry, nine studies of Asian ancestry and two studies of African-American ancestry. The majority were population-based or hospital-based case–control studies, but some studies were nested in cohorts, selected samples by age, oversampled for cases with a family history or selected samples on the basis of tumour characteristics (Table S1 in Additional file 1). Studies provided ~2% of samples in duplicate for quality control purposes (see below). Study subjects were recruited on protocols approved by the Institutional Review Boards at each participating institution, and all subjects provided written informed consent (Additional file 2).

### Genotyping and post-genotyping quality control

Genotyping for rs10235235 was carried out as part of a collaboration between the BCAC and three other consortia (the Collaborative Oncological Gene-environment Study (COGS)). Full details of SNP selection, array design, genotyping and post-genotyping quality control have been published [5]. Briefly, three categories of SNPs were chosen for inclusion in the array: SNPs selected on the basis of pooled genome-wide association study data; SNPs selected for the fine-mapping of published risk loci; and candidate SNPs selected on the basis of previous analyses or specific hypotheses. rs10235235 was a candidate SNP selected on the basis of our previous analyses [19].

For the COGS project overall, genotyping of 211,155 SNPs in 114,225 samples was conducted using a custom Illumina Infinium array (iCOGS; Illumina, San Diego, CA, USA) in four centres. Genotypes were called using Illumina’s proprietary GenCall algorithm. Standard quality control measures were applied across all SNPs and all samples genotyped as part of the COGS project. Samples were excluded for any of the following reasons: genotypically not female XX (XY, XXY or XO, *n* = 298); overall call rate <95% (*n* = 1,656); low or high heterozygosity (*P* < 10^-6^, separately for individuals of European, Asian and African-American ancestry, *n* = 670); individuals not concordant with previous genotyping within the BCAC (*n* = 702); individuals where genotypes for the duplicate sample appeared to be from a different individual (*n* = 42); cryptic duplicates within studies where the phenotypic data indicated that the individuals were different, or between studies where genotype data indicated samples were duplicates (*n* = 485); first-degree relatives (*n* = 1,981); phenotypic exclusions (*n* = 527); or concordant replicates (*n* = 2,629).

Ethnic outliers were identified by multidimensional scaling, combining the iCOGS array data with the three Hapmap2 populations, based on a subset of 37,000 uncorrelated markers that passed quality control (including ~1,000 selected as ancestry informative markers). Most studies were predominantly of a single ancestry (European or Asian), and women with >15% minority ancestry, based on the first two components, were excluded (*n* = 1,244). Two studies from Singapore (SGBCC) and Malaysia (MYBRCA; see Table S1 in Additional file 1 for all full study names) contained a substantial fraction of women of mixed European/Asian ancestry (probably of South Asian ancestry). For these studies, no exclusions for ethnic outliers were made, but principal components analysis (see below) was used to adjust for inflation in these studies. Similarly, for the two African-American studies (NBHS and SCCS), no exclusions for ethnic outliers were made.

Principal component analyses were carried out separately for the European, Asian and African-American subgroups, based on a subset of 37,000 uncorrelated SNPs. For the analyses of European subjects, we included the first six principal components as covariates, together with a seventh component derived specific to one study (LMBC) for which there was substantial inflation not accounted for by the components derived from the analysis of all studies. Addition of further principal components did not reduce inflation further. Two principal components were included for the studies conducted in Asian populations and two principal components were included for the African-American studies.

For the main analyses of rs10235235 and breast cancer risk, we excluded women from three studies (BBCS, BIGGS and UKBGS) that were genotyped in the hypothesis-generating study (*n* = 5,452) [19] and women with non-invasive cancers (ductal carcinoma *in situ*/lobular carcinoma *in situ*, *n* = 2,663) or cancers of uncertain status (*n* = 960)). After exclusions there were 47,346 invasive breast cancer case samples and 47,570 control samples from 49 studies (38 from populations of predominantly European ancestry, nine Asian and two African-American) used in the analysis (Tables S1 and S2 in Additional file 1). After quality control exclusions (above) the call rate for rs10235235 was 100% (one no call in 94,916 samples), and for the controls there was no evidence of deviation from Hardy–Weinberg equilibrium in any of the contributing studies (Table S2 in Additional file 1).

We did not test for an association between rs10235235 and age at menarche in our hypothesis-generating study [19]. Therefore, to maximise our power to detect an association, we included menarche data from BBCS cases (*n* = 2,508) and controls (*n* = 1,650) and from UKBGS cases (*n* = 3,388) and controls (*n* = 4,081) in this analysis. Age at menarche was not available for samples from BIGGS. Full details of genotyping of rs10235235 in BBCS and UKBGS samples have been published previously [19]. Briefly, genotyping was carried out using competitive allele-specific polymerase chain reaction KASPar chemistry (KBiosciences Ltd, Hoddesdon, Hertfordshire, UK). Call rates were 98.0% (BBCS) and 96.6% (UKBGS); there was no evidence for deviation from Hardy–Weinberg equilibrium (*P* = 0.29 (BBCS); *P* = 0.92 (UKBGS)), and the duplicate concordance based on a 1% (BBCS) and 5% (UKBGS) random sample of duplicates was 100% for both studies.

### Statistical analysis

We estimated per-allele and genotypic log odds ratios (ORs) for the European, Asian and African-American subgroups separately using logistic regression, adjusted for principal components and study [5]. To test for departure from a multiplicative model we compared multiplicative and unconstrained models using a one degree of freedom likelihood ratio test. Heterogeneity in ORs between studies within each subgroup (European, Asian and African-American), and between subgroups, was assessed using the Cochrane *Q* statistic and quantified using the *I*^*2*^ measure [20].

Analyses stratified by oestrogen receptor status (+/–), progesterone receptor status (+/–), morphology (ductal or lobular), grade (1,2,3), lymph node involvement (+/–) or age at diagnosis (≤50 and >50 years) were restricted to studies of European ancestry due to the small number of studies of Asian and African-American ancestry. In addition, studies were excluded if they had selected cases on the basis of the stratifying variable, or had collected data on that variable for less than 5% of cases or less than 10 cases in total. Availability of data for each of the stratifying variables in each study is shown in Table S3 in Additional file 1. To assess the relationship between each of the stratifying variables and genotype, stratum-specific ORs were calculated using logistic regression. Cases in each stratum were compared with all control subjects, adjusted for study and principal components. Case-only logistic regression was used to test for heterogeneity between strata (binary stratifying variables) or across strata (stratifying variables with three or more strata). *P* values were estimated using likelihood ratio tests with one degree of freedom.

We assessed whether rs10235235 was associated with age at menarche in cases and controls separately. Studies that had not collected data on age at menarche in both cases and controls were excluded (Table S4 in Additional file 1). We used linear regression, adjusted for principal components and study, to estimate the relationship between age at menarche (years) and rs10235235 genotype (0, 1, 2 rare alleles) and logistic regression adjusted for principal components and study to estimate the association between age at menarche and breast cancer risk. To test for effect modification of an association between rs10235235 and breast cancer risk by age at menarche, we used logistic regression adjusted for principal components, study and age at menarche (grouped as ≤11, 12, 13, 14 and ≥15 years) with and without an interaction term(s). We considered four models: no interaction (zero interaction terms); assuming a linear interaction between genotype and menarche group (one interaction term); assuming a linear interaction between genotype and menarche group but allowing the linear term to differ between women who were heterozygous and those who were homozygous for the rare allele (two interaction terms); and one interaction term for each possible genotype/menarche group combination (eight interaction terms). Nested models were compared using likelihood ratio tests. All statistical analyses were performed using STATA version 11.0 (StataCorp, College Station, TX, USA). All *P* values reported are two-sided.

## Results

The case–control analysis comprised genotype data for 47,346 invasive breast cancer cases and 47,569 controls from 49 studies, including 80,518 (84.8%) subjects of self-reported European ancestry, 12,419 (13.1%) of self-reported Asian ancestry and 1,978 (2.1%) of self-reported African-American ancestry. The mean (± standard deviation) age at diagnosis was 56.1 (± 11.6) years for European cases, 51.1 (± 10.5) years for Asian cases and 53.1 (± 10.7) years for African-American cases. There were ethnic differences in the estimated minor allele frequency (MAF) of rs10235235 (*Q* = 7317.1, two degrees of freedom; *P* for heterogeneity (*P*_het_) = 0). The overall MAF for European control women was 0.089 (95% CI = 0.087, 0.091), but with strong evidence of between-study heterogeneity (*P*_het_ = 1 × 10^-22^) that was accounted for by the three Finnish studies (HEBCS, MAF = 0.15; KBCP, MAF = 0.21; and OBCS, MAF = 0.15; *P*_het_ = 0.01); no evidence of heterogeneity remained after taking account of these studies (MAF = 0.087 (95% CI = 0.085, 0.089); *P*_het_ = 0.23). Relative to Europeans, the overall MAF was higher for African-Americans (0.213, 95% CI = 0.195, 0.232; *P*_het_ = 0.26) but much lower for Asians (0.002; 95% CI = 0.001, 0.002), with strong evidence of between-study heterogeneity for the latter (*P*_het_ = 4 × 10^-14^).

**Figure 1:**
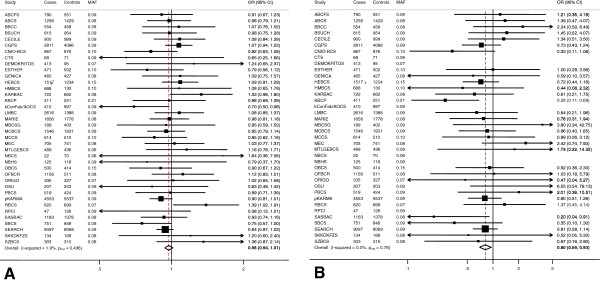
**Association of rs10235235 with breast cancer risk for women of European ancestry.** Forest plots of the association of the rs10235235 AG (heterozygote) genotype **(A)** and GG (homozygote) genotype **(B)** with breast cancer risk for women of European ancestry. Horizontal lines, 95% confidence intervals (CIs); square boxes, study-specific fixed-effects estimates; diamond, combined, fixed-effects estimate of the odds ratio (OR) and 95% CI. Vertical line, null effect (OR = 1.0); dashed vertical line, estimated heterozygote OR **(A)** and estimated homozygote OR **(B)**. Homozygote ORs for six studies (CTS, DEMOKRITOS, kConFab/AOCS, NBCS, NBHS and RPCI) could not be estimated because there were no GG homozygotes among cases or among controls in each of these studies (see Table S2 in Additional file 1).

The case–control analysis was consistent with a modest association between rs10235235 and breast cancer risk for women of European ancestry, with an estimated per-allele OR of 0.96 (95% CI = 0.93, 0.99; *P* for linear trend (*P*_trend_) = 0.02). Genotype-specific ORs were 0.98 (95% CI = 0.94, 1.01; *P* = 0.21) for AG versus AA (Figure 1A) and 0.80 (95% CI = 0.69, 0.93; *P* = 0.004) for GG versus AA (Figure 1B), with no evidence of between-study heterogeneity for either OR estimate (*P*_het_ = 0.44, *I*^*2*^ = 1.9% and *P*_het_ = 0.76*, I*^*2*^ = 0.0% for heterozygote and homozygote OR estimates respectively). There was, however, marginally significant evidence that the genotypic OR estimates departed from those expected under a multiplicative model with the inverse association of the GG genotype being more than the square of that of the AG genotype (test for deviation from multiplicative model, *P* = 0.04).

Data for rs10235235 in women of Asian or African-American ancestry were more limited, with just two African-American studies (1,046 cases and 932 controls) and nine Asian studies (5,795 cases and 6,624 controls). In addition, this SNP was sufficiently rare in Asian populations (MAF = 0.002) that we were unable to estimate the heterozygote OR in two Asian studies (SEBCS, one carrier among 1,114 cases and no carriers among 1,129 controls; TWBCS, one carrier among 236 controls and no carriers among 774 cases; Table S2 in Additional file 1) and we could not estimate a homozygote OR for any Asian study (Table S2 in Additional file 1). There was no clear evidence that this SNP was associated with breast cancer risk for women of Asian ancestry (heterozygote OR = 1.06, 95% CI = 0.76, 1.49) or African-American ancestry (heterozygote and homozygote ORs were OR = 1.09, 95% CI = 0.90, 1.32 and OR = 0.94, 95% CI = 0.62, 1.42 respectively; Figure S1 in Additional file 1). This analysis, however, had low power to detect associations in non-Europeans and these OR estimates were not inconsistent with the magnitude of the observed OR estimates for European women (*P*_het_ = 0.51).

Stratifying cases by oestrogen receptor (*P*_het_ = 0.83) or progesterone receptor (*P*_het_ = 0.19) status, tumour grade (*P*_het_ = 0.63) or nodal involvement at diagnosis (*P*_het_ = 0.51) showed no evidence of effect modification (Table 1). There was some evidence of effect modification by morphology (*P*_het_ = 0.03). For ductal cancers we estimated a very modest reduction of risk for heterozygotes (OR_het_ = 0.98, 95% CI = 0.93, 1.02; *P* = 0.30) and a stronger, significant reduction for homozygotes (OR_hom_ = 0.74, 95% CI = 0.61, 0.90; *P* = 0.003). For lobular cancers there was no such trend (OR_het_ = 1.07, 95% CI = 0.98, 1.17; *P* = 0.14 and OR_hom_ = 0.91, 95% CI = 0.64, 1.27; *P* = 0.57).

**Table 1:** Association of rs10235235 with risk of breast cancer for women of European ancestry: stratified analysis

	**Cases**	**Controls**	**OR**_**het**_	**95% CI**	***P***_**1**_	**OR**_**hom**_	**95% CI**	***P***_**1**_	***P***_**het**_
ER status									
ER-positive	24,780	38,739	0.99	0.95, 1.03	0.61	0.83	0.70, 0.99	0.04	
ER-negative	5,851	38,739	1.02	0.95, 1.10	0.60	0.60	0.43, 0.86	0.005	
NK	8,339								
Total	38,970^a^	38,739	0.99	0.95, 1.03	0.74	0.79	0.67, 0.94	0.006	0.83
PR status									
PR-positive	18,497	39,033	0.98	0.93, 1.02	0.32	0.82	0.67, 0.99	0.04	
PR-negative	8,193	39,033	1.02	0.96, 1.09	0.53	0.74	0.56, 0.98	0.03	
NK	12,111								
Total	38,801^b^	39,033	0.99	0.94, 1.03	0.52	0.80	0.67, 0.95	0.01	0.19
Morphology									
Ductal	22,123	31,803	0.98	0.93, 1.02	0.30	0.74	0.61, 0.90	0.003	
Lobular	3,921	31,803	1.07	0.98, 1.17	0.14	0.91	0.64, 1.27	0.57	
Other and NK	5,995								
Total	32,039	31,803	0.99	0.95, 1.04	0.64	0.77	0.64, 0.92	0.004	0.03
Grade									
Grade 1	5,944	37,285	0.97	0.90, 1.05	0.46	0.86	0.65, 1.15	0.31	
Grade 2	13,427	37,285	1.00	0.95, 1.06	0.92	0.80	0.63, 0.98	0.04	
Grade 3	8,638	37,285	0.98	0.92, 1.05	0.58	0.61	0.46, 0.82	0.001	
NK	8,769								
Total	36,778	37,285	0.99	0.95, 1.03	0.56	0.76	0.64, 0.90	0.001	0.63
Nodal status									
Node-negative	17,463	37,836	0.98	0.93, 1.03	0.47	0.86	0.71, 1.04	0.12	
Node-positive	10,746	37,836	0.98	0.92, 1.04	0.46	0.72	0.57, 0.93	0.01	
NK	9,359								
Total	37,568	37,836	0.98	0.94, 1.02	0.31	0.81	0.68, 0.96	0.02	0.51

Association of rs10235235 with risk of breast cancer for women of European ancestry stratified by oestrogen receptor (ER) status, progesterone receptor (PR) status, morphology, grade and nodal status. OR_het_, odds ratio comparing rs10235235 AG genotype versus AA genotype; H_0_, null hypothesis; NK, not known; OR_hom_, odds ratio comparing rs10235235 GG genotype versus AA genotype; *P*_1_, test of H_0_ no association between rs10235235 and breast cancer risk; *P*_het_, test of H_0_ no difference between stratum specific estimates for variables with two strata or test of H_0_ no linear trend in stratum specific estimates for variables with three strata. ^a^Excludes seven studies that selected all ER-negative cases (CTS, DEMOKRITOS, NBCS, NBHS, OSU, RPCI and SKKDKFZS) and one study (PBCS) that selected all ER-positive cases. ^b^Excludes seven studies that selected all PR-negative cases (CTS, DEMOKRITOS, NBCS, NBHS, OSU, RPCI and SKKDKFZS).

The SNP rs10235235 maps to a locus (*CYP3A*) that has been considered an *a priori* candidate for involvement in determining age at menopause and age at menarche [21,22]. Stratifying cases by age at diagnosis (≤50 or >50 years) as a proxy for menopausal status at diagnosis showed no evidence of effect modification (*P*_het_ = 0.89; Table 2), and excluding cases who were diagnosed between age 46 and 55 as potentially perimenopausal did not alter this result (*P*_het_ = 0.28). Data on age at menarche were available for 21,736 cases and 22,686 controls (Table S4 in Additional file 1); to increase the power of the analysis we included additional data from BBCS and UKBGS (5,737 cases, 5,572 controls; Table S4 in Additional file 1) [19]. There was a 1.5% (95% CI = 0.5%, 2.7%; *P* = 0.004) reduction in breast cancer risk associated with each additional year’s increase in age at menarche. Mean age at menarche was positively associated with number of copies of the minor allele of rs10235235 for controls (*P*_trend_ = 0.005; Table 3) but not for cases (*P*_trend_ = 0.97; Table 3). Consequently, there was an inverse trend in the magnitude of the heterozygote and homozygote breast cancer ORs with mean age at menarche (*P*_het_ = 0.02; Table 4); being a carrier of one or two rare alleles of rs10235235 was associated with an estimated 16% (OR_het_ = 0.84, 95% CI = 0.75, 0.94; *P* = 0.003) or 19% (OR_hom_ = 0.81, 95% CI = 0.51, 1.30; *P* = 0.39) (*P*_trend_ = 0.002) reduction in breast cancer risk for women who had their menarche at ages ≥15 years but there was no evidence of reduction for those with a menarche at age ≤11 years (OR_het_ = 1.06, 95% CI = 0.95, 1.19; *P* = 0.30 and OR_hom_ = 1.07, 95% CI = 0.67, 1.72; *P* = 0.78) (*P*_trend_ = 0.29). There was no evidence that the inverse trend in the magnitude of ORs with mean age at menarche differed between heterozygous and homozygous carriers (*P* = 0.97) and no evidence that the trend was nonlinear (*P* = 0.70).

**Table 2:** rs10235235 and risk of breast cancer for women of European ancestry by age at diagnosis

**Age at diagnosis**	**Cases**^**a**^	**Controls**^**a**^	**OR**_**het**_	**95% CI**	***P***_**1**_	**OR**_**hom**_	**95% CI**	***P***_**1**_	***P***_**het**_
≤ 50 years	11,794	34,988	0.99	0.93, 1.05	0.69	0.68	0.53, 0.86	0.003	
> 50 years	23,264	34,988	0.97	0.93, 1.02	0.24	0.84	0.70, 1.00	0.04	
NK	554								
Total	35,612	34,988	0.98	0.94, 1.02	0.23	0.79	0.67, 0.92	0.003	0.89

^a^Five studies (ABCFS, MARIE, MEC, MTLGEBCS and SASBAC) that selected all cases on the basis of age at diagnosis (Table S3 in Additional file 1) were excluded from this stratified analysis; two small studies (CTS and NBCS) that had no heterozygote or rare homozygote cases in one of the age stratum were also excluded. H_0_, null hypothesis; NK, not known; OR_het_, odds ratio comparing rs10235235 AG genotype versus AA genotype; OR_hom_, odds ratio comparing rs10235235 GG genotype versus AA genotype; *P*_1_, test of H_0_ no association between rs10235235 and breast cancer risk; *P*_het_, test of H_0_ no difference between stratum specific estimates.

**Table 3:** Association of rs10235235 with age at menarche for women of European ancestry by case-control status

**rs10235235 genotype**	**Cases**	**Age at menarche (years)**	***P***_**trend**_	**Controls**	**Age at menarche (years)**	***P***_**trend**_
AA	22,954	12.83		23,383	12.95	
AG	4,312	12.83		4,627	13.02	
GG	207	12.83		248	13.05	
Total	27,473	12.83	0.97	28,258	12.96	0.005

H_0_, null hypothesis; *P*_trend_, test of H_0_ no linear trend in age at menarche according to rs10235235 genotype.

**Table 4:** rs10235235 and risk of breast cancer for women of European ancestry by age at menarche

**Age at menarche (years)**	**Cases**	**Controls**	**OR**_**het**_	**95% CI**	***P***_**1**_	**OR**_**hom**_	**95% CI**	***P***_**1**_	***P***_**het**_
≤11	4,818	4,749	1.06	0.95, 1.19	0.30	1.07	0.67, 1.72	0.78	
12	5,655	5,720	0.92	0.83, 1.02	0.10	0.83	0.54, 1.28	0.41	
13	7,308	7,379	0.93	0.85, 1.02	0.11	0.77	0.54, 1.09	0.14	
14	5,307	5,743	0.96	0.86, 1.06	0.42	0.69	0.45, 1.06	0.09	
≥15	4,385	4,667	0.84	0.75, 0.94	0.003	0.81	0.51, 1.30	0.39	
Total	27,473	28,258	0.94	0.90, 0.98	0.007	0.81	0.67, 0.98	0.03	0.02

H_0_, null hypothesis; OR_het_, odds ratio comparing rs10235235 AG genotype versus AA genotype; OR_hom_, odds ratio comparing rs10235235 GG genotype versus AA genotype; *P*_1_, test of H_0_ no association between rs10235235 and breast cancer risk; *P*_het_, test of H_0_ no linear trend in stratum specific estimates.

## Discussion

This study of more than 47,000 breast cancer cases and 47,000 controls has confirmed that rs10235235, mapping to 7q22.1 (*CYP3A*), is associated with a reduction in breast cancer risk for women of European ancestry. Previously, our hypothesis-generating study of 10,000 breast cancer cases and 17,000 controls found a per-allele OR estimate of 0.96 (95% CI = 0.90, 1.02; *P* = 0.2), with marginally significant evidence of an inverse association for breast cancer diagnosed age 50 years or younger (OR = 0.91, 95% CI = 0.83, 0.99; *P* = 0.03) but no evidence of an association for breast cancer at later ages (OR = 1.01, 95% CI = 0.93, 1.10; *P* = 0.82) [19]. In this considerably larger study, we found a heterozygote OR estimate of 0.98 (95% CI = 0.94, 1.01; *P* = 0.21) and a homozygote OR estimate of 0.80 (95% CI = 0.69, 0.93; *P* = 0.004) with marginally significant evidence that the inverse association for homozygotes is greater than predicted by a multiplicative model (*P* = 0.04).

To our knowledge, rs10235235 is the first SNP to be associated with both breast cancer risk and age at menarche, consistent with the well-documented association between later age at menarche and a reduction in breast cancer risk [23]. Genome-wide association studies have identified more than 70 breast cancer risk variants [5,6] and more than 30 variants associated with age at menarche [22], none of which map to the *CYP3A* locus. rs10235235 was originally identified on the basis of a highly significant association with hormone levels, accounting for 4.9% of the variation in premenopausal urinary oestrone glucuronide levels [19]. In this current analysis, rs10235235 accounted for only 0.01% of the variation across controls in age at menarche and we estimate that this SNP explains just 0.01% of the familial excess breast cancer risk. Our data thus illustrate the potential statistical efficiency of studies of intermediate phenotypes in the identification of rarer (MAF < 10%) risk alleles with modest associations. Our analysis shows some inconsistency with a recent genome-wide study of circulating oestradiol, testosterone and sex hormone-binding globulin in postmenopausal women [24]. In that study there was no genome-wide significant association observed with plasma oestradiol levels in either the primary analysis of approximately 1,600 postmenopausal women who were not taking postmenopausal hormones at blood draw or the secondary analysis that included approximately 900 current postmenopausal hormone users. Further studies will be needed to determine whether the lack of an association between *CYP3A* variants and postmenopausal plasma oestradiol levels reflects a difference in the menopausal status of the study subjects, the hormone/metabolite that was analysed or chance.

One possible explanation for the apparent effect modification of the rs10235235–breast cancer risk association by age at menarche is that this is a function of genotyping a marker SNP rather than the true causal variant. For example, if rs10235235 was perfectly correlated with a causal variant, SNP X, with a MAF substantially lower than that of rs10235235 (*D*′ ~ 1.0, *r*^2^ < 1.0), then there would be three types of chromosome in the population: type i, chromosomes carrying the common allele of rs10235235 and the common allele of SNP X; type ii, chromosomes carrying the rare allele of rs10235235 and the common allele of SNP X; and type iii, chromosomes carrying the rare allele of rs10235235 and the rare (protective) allele of SNP X. Only chromosomes carrying the rare allele of rs10235235 and the rare (protective) allele of SNP X (type iii) would be enriched in controls. Genotyping the marker (rs10235235) rather than the causal variant leads to misclassification. As the causal variant is associated with a protective effect on breast cancer risk, the proportion of chromosomes carrying both the rare allele of the causal variant and the marker (type iii) compared with the common allele of the causal variant and the rare allele of the marker (type ii) will be greater in controls than in cases such that the extent of misclassification will be greater for cases than controls. This will attenuate the association between genotype and age at menarche to a greater extent in cases than in controls creating an apparent effect modification. Fine mapping and functional studies will be required to identify the causal variant and to determine the true relationship between the causal variant, age at menarche and breast cancer risk.

Despite our original finding of a strong association between rs10235235 and hormone levels, we found no evidence that the association between this SNP and breast cancer risk differed by the hormone receptor status of the tumour, and nor did we find any evidence that the association differed by stage, grade or lymph node involvement. There was marginally significant evidence that the association between rs10235235 and breast cancer risk differed between ductal and lobular cancers (*P*_het_ = 0.03). Given the number of stratified analyses that we carried out (six stratifying variables) and given that there is no biological basis to support an interaction between rs10235235 and morphology, this is probably a chance observation.

In contrast to our earlier study [19], we found no evidence of an interaction with age at diagnosis when we stratified cases by age ≤/>50 years, either including or excluding cases diagnosed between age 46 and 55 years as potentially perimenopausal. We used age at diagnosis as a proxy for menopausal status at diagnosis because menopausal status at diagnosis is difficult to determine by questionnaire, especially given the use of hormone replacement therapies; while information on age at diagnosis was available for all but 1.4% (*n* = 554) of cases, information on age at natural menopause was missing for 65.6% (*n* = 26,552) of cases of European ancestry. Similarly, although rs10235235 is a plausible candidate for association with age at menopause, we did not test this due to the limited amount of data on age at natural menopause for controls of European ancestry (*n* = 11,294, 28.2%) and the difficulty in ascertaining whether treatment for breast cancer had influenced reported age at menopause for cases.

The strengths of our study include the large size of this combined analysis, and the availability of information on tumour characteristics for the majority of cases and on age at menarche for the majority of cases and controls. Limitations include low power of the study to examine an association between genotype and breast cancer risk for non-Europeans.

## Conclusions

In summary, we have confirmed that rs10235235 is associated with breast cancer, have shown for the first time that rs10235235 is associated with age at menarche in controls and have suggested a potential mechanism for these associations. rs10235235, which maps to the *CYP3A* locus, probably tags a causal variant that affects expression of one or more *CYP3A* genes.

## Abbreviations

BCAC: Breast Cancer Association Consortium; CI: confidence interval; COGS: Collaborative Oncological Gene-environment Study; MAF: minor allele frequency; OR: odds ratio; *P*_trend_: *P* value for linear trend; SNP: single nucleotide polymorphism.

## Competing interests

The authors state that they have no competing interests.

## Authors’ contributions

OF, FD and NO performed the statistical analyses. OF, IdSS and NJ drafted the manuscript. NJ, FD, NO, LG, MEJ, MJS, EJF, BPH, MG-C, MDo, AA, AJS, JP, IdSS and OF comprised the writing group that was responsible for the interpretation of the results and for critically reviewing the manuscript. AC, AJ, AHW, AMa, BBu, C-YS, DL, ES, GC-T, HN, HBre, HBra, ILA, JC-C, J-YC, JLH, LBa, MKB, HMi, PAF, PR, RW, SEB, TD, MKS and UH also significantly contributed to the interpretation of the results. OF, IdSS, NJ, JP, LG, DFE, MKB and JW conceived of the original design of the study and participated in subject recruitment and in acquisition of data. JBen, AG-N, RM, DCT, DV, FB, CL, JD, JS and KMi carried out the genotyping and/or data analysis. FD, NO, MEJ, MJS, EJF, BPH, JLH, MCS, GSD, CA, MKS, AB, LJVV, FA, KMu, ALo, PAF, MWB, ABE, SPR, ES, IT, MK, NM, BBu, FMa, AS, CS, PG, TT, EC, FMe, SEB, BGN, HF, RMi, MPZ, JIAP, JBen, LBe, HA-C, AZ, CCD, HBre, HMü, VA, AKD, AMe, JH, CRB, RKS, HBra, CJ, Y-DK, The GENICA Network, HN, TAM, KA, CB, KMa, TD, NVB, NNA, ALi, AMa, VK, V-MK, JMH, GC-T, JBee, kConFab Investigators, Australian Ovarian Cancer Study Group, AHW, DVdB, C-CT, DL, DS, PN, HW, JC-C, AR, SN, DF-J, PR, PP, BBo, VP, FJC, JEO, XW, ZF, VSP, GGG, GS, LBa, CH, JS, MSG, FL, MDu, PS, ST, CHY, SYP, BKC, VNK, GGA, A-LB-D, WZ, RW, KP, AJ-V, MG, ILA, JAK, GG, AMM, PD, JF, SJC, JLis, MES, PH, NS, MHo, AH, RAO, MT-L, JLiu, AC, IWB, MWRR, SSC, WB, LBS, PDPP, AMD, MS, DK, D-YN, SKP, J-YC, MHa, HMi, WYL, AT, UH, AF, TR, HUU, AJ, JLu, KJ-B, KD, SSa, VG, PB, JM, SSl, AET, CV, DY, C-YS, J-CY, C-SH, M-FH, AG-N, DCT, DV, FB, CL, JD, KMi, MKB, JW, DFE, MG-C, MDo, AA and AJS made substantial contributions in recruiting subjects and acquiring data, and in critically reviewing the manuscript. All authors take responsibility for the work and read and approved the final version of the manuscript.

## Supplementary Material

Additional file 1 Contains Table S1 presenting details of participating BCAC studies; Table S2 presenting rs10235235 genotypes for breast cancer cases and controls from 49 BCAC studies; Table S3 presenting availability of data on age at diagnosis, hormone receptor status, morphology, grade and nodal status for breast cancer cases from 38 European BCAC studies; Table S4 presenting availability of data on age at menarche for breast cancer cases and controls from 40 European BCAC studies; and Figure S1 showing association of the rs10235235-AG genotype with breast cancer risk for women of Asian and African-American ancestry. Click here for file

Additional file 2 Presents details of ethical committees that approved each study. Click here for file
