# Supplementary material for: Genetic variation at CYP3A is associated with age at menarche and breast cancer risk: a case-control study
Source: Breast Cancer Res. 2014 May 26;16(3):R51. doi: 10.1186/bcr3662 (PMC4522594; doi:10.1186/bcr3662)
Supplement: Additional file 1 — Contains Table S1 presenting details of participating BCAC studies; Table S2 presenting rs10235235 genotypes for breast cancer cases and controls from 49 BCAC studies; Table S3 presenting availability of data on age at diagnosis, hormone receptor status, morphology, grade and nodal status for breast cancer cases from 38 European BCAC studies; Table S4 presenting availability of data on age at menarche for breast cancer cases and controls from 40 European BCAC studies; and Figure S1 showing association of the rs10235235-AG genotype with breast cancer risk for women of Asian and African-American ancestry. [file bcr3662-S1.zip › Additional file 1/4703570011029251_add1.docx]

**Additional file 1**

1. **Table S1** **Details of participating BCAC studies**
2. **Table S2** **rs10235235 genotypes for breast cancer cases and controls from 49 BCAC studies**
3. **Table S3 Availability of data on age at diagnosis, hormone receptor status, morphology, grade and nodal status for breast cancer cases from 38 European BCAC studies**
4. **Table S4** **Availability of data on age at menarche for breast cancer cases and controls from 40 European BCAC studies**
5. **Figure S1 Association of rs10235235-AG genotype with breast cancer risk for women of Asian and African-American ancestry**

**Table S1** **Details of participating BCAC studies**

| **Study Acronym** | **Study Name** | **Country** | **Recruitment base** | | |
| --- | --- | --- | --- | --- | --- |
|  |  |  | **Cases** | **Controls** | |
| **STUDIES OF WHITE EUROPEAN WOMEN** | | | | | |
| ABCFS | Australian Breast Cancer Family Study[1] | Australia | Cancer registries in Victoria and New South Wales (1992-1999): all cases from Melbourne and Sydney diagnosed before age 40 plus a random sample of those diagnosed at ages 40-59. | | Identified between 1992-1999 from the electoral rolls in Melbourne and Sydney (enrolling to vote is compulsory); frequency matched to cases by age in 5 year categories. |
| ABCS | Amsterdam Breast Cancer Study[2] | Netherlands | Breast cancer patients diagnosed before age 50 in 2003-2009 at the NKI-AVL; and (ABCS-F) All non-BRCA1/2 breast cancer cases from the family cancer clinic of the NKI-AVL tested in the period 1995-2009; all ages and diagnosed with breast cancer in 1965-2008. | | Population-based cohort of women recruited through the Sanquin blood bank, all ages. |
| BBCC | Bavarian Breast Cancer Cases and Controls[3] | Germany | Consecutive, unselected cases with invasive breast cancer recruited at the University Breast Centre, Franconia in Northern Bavaria from 2002-2010. | | Healthy women aged 55 or older with no diagnosis of cancer. Invited by a newspaper advertisement in Northern Bavaria between 2002-2010. |
| BBCS | British Breast Cancer Study[4] | UK | (i) English & Scottish Cancer Registries: all breast cancer cases who developed a first primary before age 66 in 1971 or later and who subsequently developed a second primary cancer. (ii) Breast Cancer Clinics: all breast cancer cases who developed a first primary before age 71 in 1967 or later and who either subsequently developed a second primary or had at least two affected female first-degree relatives.  All recruited from 2001-2008. | | A friend, sister-in-law, daughter-in-law or other non-blood relative of cases, recruited from 2001-2008. |
| BIGGS | Breast Cancer in Galway Genetic Study[5] | Ireland | Unselected cases recruited from University College Hospital Galway and surrounding hospitals in the West of Ireland since 2001 | | Women > 60 years with no personal history of any cancer and no family history of breast or ovarian cancer identified from retirement groups in the West of Ireland between 2001-2008. |
| BSUCH | Breast Cancer Study of the University Clinic Heidelberg[6] | Germany | All cases diagnosed with breast cancer in 2007-2009 at the University Women`s Clinic Heidelberg. | | Female blood donors recruited in 2007- 2009 at the Institute of Transfusion Medicine & Immunology, Mannheim. |
| CECILE | CECILE Breast cancer study[7] | France | All cases diagnosed with breast cancer in 2005-2007 among women <75 years of age residing in the *départements* of Ille-et-Vilaine and Côte d'Or. Cases were recruited from the main cancer treatment center (Centre Eugène-Marquis in Rennes and Centre Georges-François-Leclerc in Dijon) and from other private or public hospitals in each area. | | General population control women residing in the same areas as the cases (Ille-et-Vilaine and Côte d’Or). Controls were frequency-matched to the cases by 5-year age groups. They were recruited in 2005-2007 using a random digit dialing procedure and quotas by socioeconomic status to reflect the distribution by SES of the population in each area. |
| CGPS | Copenhagen General Population Study[8] | Denmark | Consecutive, incident cases from one hospital with centralized care for a population of 400,000 women in Copenhagen (2001-present). | | Women with no history of breast cancer residing in the same region as cases identified from the Copenhagen General Population Study (2003-2007). |
| CNIO-BCS | Spanish National Cancer Centre Breast Cancer Study[9] | Spain | (i) consecutive breast cancer patients from three public hospitals, two in Madrid and one in Oviedo;  (ii) cases with at least one affected first degree relative recruited through the CNIO family cancer clinic in Madrid (2000-2005). | | Women attending the Menopause Research Centre, Madrid and female members of the College of Lawyers attending a free, targeted medical check-up in Madrid, all free of breast cancer and all in Madrid between 2000-2005. |
| CTS* | California Teachers Study[10] | USA | Nested case-control study conducted within a cohort of California teachers (113,590) who were under age 80 years at baseline, had no prior history of invasive or *in situ* breast cancer. Cases are women newly diagnosed with a histologically confirmed invasive primary adenocarcinoma of the breast at age 80 years or younger from 1998 to 2008. | | Controls are a probability sample of at-risk cohort members, frequency matched to cases on age at baseline (5-year age groups), self-reported race/ethnicity (white, African American, Latina,Asian, other), and broad geographic region within California.  Controls were selected without replacement, using an assigned reference date. |
| DEMOKRITOS* | DEMOKRITOS | Greece | Triple negative breast cancer cases enrolled from 1997-2010 in hospitals serving geographical areas of Greece, including Athens metropolitan area, Thessaloniki, Ioannina, Patras, and Crete (Chania), in collaboration with the Hellenic Cooperative Oncology Group (HECOG). | | Regional controls from Athens, Greece were population-based unaffected women of the same age range. |
| ESTHER | ESTHER Breast Cancer Study[11] | Germany | Breast cancer cases in all hospitals in the state of Saarland, from 2001-2003 (ESTHER) and 1996-1998 (VERDI). | | Random sample of women from a routine health check-up in Saarland, in 2000-2002; frequency matched to cases by age in-5 year categories. |
| GC-HBOC | German Consortium for Hereditary Breast and Ovarian Cancer[12] | Germany | BRCA1/2 mutation negative cases from University Clinics in Cologne and Munich | | KORA (Cooperative Health Research in the Region Augsburg) |
| GENICA | Gene Environment Interaction & Breast Cancer in Germany[12, 13] | Germany | Incident breast cancer cases enrolled at hospitals in the Greater Bonn area between 2000-2004. | | Random address sample selected in 2001-2004 from 31 population registries in the greater Bonn area; frequency matched to cases on year of birth in 5-year categories. |
| HEBCS | Helsinki Breast Cancer Study | Finland | (1) Consecutive cases (883) from the Department of Oncology, Helsinki University Central Hospital 1997-8 and 2000, (2) Consecutive cases (986) from the Department of Surgery, Helsinki University Central Hospital 2001 – 2004, (3) Familial breast cancer patients (536) from the Helsinki University Central Hospital, Departments of Oncology and Clinical Genetics (1995-). | | Healthy females from the same geographical region in Southern Finland in 2003. |
| HMBCS | Hannover-Minsk Breast Cancer Study[14] | Belarus | Cases from the Byelorussian Institute for Oncology and Medical Radiology Aleksandrov N.N. in Minsk or at one of 5 regional oncology centers in Gomel, Mogilev, Grodno, Brest or Vitebsk (2002-2008). | | Women attending general medical examination at gynecology clinics in Gomel, Mogilev, Grodno, Brest or Vitebsk; women attending the Institute for Inherited Diseases in Minsk; female blood donors in Minsk; healthy relatives of cases (2002-2008). |
| KARBAC | Karolinska Breast Cancer Study | Sweden | 1. Familial cases from Department of Clinical Genetics, Karolinska University Hospital , Stockholm.  2. Consecutive cases from Department of Oncology, Huddinge & Söder Hospital, Stockholm 1998-2000. | | Blood donors of mixed gender from same geographical region. Excess material was received from all blood donors over a 3 month period in 2004 (approximately 3000) and DNA was extracted from a random sample of 1500. |
| KBCP | Kuopio Breast Cancer Project[15] | Finland | Women seen at Kuopio University Hospital between 1990-1995 because of a breast lump, mammographic abnormality, or other breast symptom and who were found to have breast cancer. | | Selected from the National Population Register between 1990-1995; age and long-term area-of-residence matched to cases. |
| kConFab/  AOCS | Kathleen Cuningham Foundation Consortium for Research into Familial Breast Cancer / Australian Ovarian Cancer Study[16] | Australia | Index (youngest affected) cases from *BRCA1*- and *BRCA2*-mutation-negative multiple-case breast and breast-ovarian families recruited though family cancer clinics from across Australia and New Zealand from 1998-present. | | Identified from the electoral rolls from across Australia as part of the Australian Ovarian Cancer Study in 2002-2006. |
| LMBC | Leuven Multidisciplinary Breast Centre[17] | Belgium | All patients diagnosed with breast cancer and seen in the Multidisciplinary Breast Center in Leuven (Gashuisberg) since June 2007 plus retrospective collection of cases diagnosed since 2000. | | Blood donors at Gasthuisberg Hospital (2007-2008). |
| MARIE | Mammary Carcinoma Risk Factor Investigation[18] | Germany | Incident cases diagnosed from 2001-2005 in the study region Hamburg in Northern Germany, and from 2002-2005 in the study region Rhein-Neckar-Karlsruhe in Southern Germany. | | 2 controls per case were randomly drawn from population registries and frequency matched by birth year and study region to the case. Controls were recruited from 2002 to 2006. |
| MBCSG | Milan Breast Cancer Study Group[19] | Italy | Familial and/or early onset breast cancer patients (aged 22-87) negative for mutations in *BRCA1* and *BRCA2*, ascertained at two large cancer centers in Milan from 2000-present. | | Female blood donors recruited at two centres in Milan from 2004-present and 2007-present. |
| MCBCS | Mayo Clinic Breast Cancer Study[20] | USA | Incident cases residing in 6 states (MN, WI, IA, IL, ND, SD) seen at the Mayo Clinic in Rochester, MN from 2002-2010. | | Women presenting for general medical examination at the Mayo Clinic from 2002-2010; frequency matched to cases on age, ethnicity and county/state. |
| MCCS | Melbourne Collaborative Cohort Study[21] | Australia | Incident cases from the cohort of 24,469 women, diagnosed during the follow-up from baseline (1990-1994) to 2008. | | Random sample of the initial cohort. |
| MEC | Multiethnic Cohort | USA | Incident cases identified from SEER cancer registries in Los Angeles County & State registries in California & Hawaii, USA from 1993-2002. Grouped by self-reported ethnicity. | | Women without cancer from the same States, recruited concurrently with cases & frequency matched to cases by age at blood-draw & self-reported ethnicity. |
| MTLGEBCS | Montreal Gene-Environment Breast Cancer Study | Canada | All cases are postmenopausal women (47-75 years) living in Montreal with a primary invasive breast cancer and with no previous occurrence of any type of cancer. All cases were identified from 2007 to 2010 in 15 of 16 Montreal hospitals that treat breast cancer. | | Random sample from the universal Provincial Voter Registration List, approximately frequency-matched to cases on age (5-year bins) and living in Montreal. |
| NBCS* | Norwegian Breast Cancer Study[22] | Norway | Incidence cases from three different hospitals: Ullevål Univ. Hospital 1990-94, Norwegian Radium Hospital 1975-1986 and 1995-1998, Haukeland Univ. Hospital 1992-2001. | | Women residing in Tromsø and Bergen who attended the Norwegian Breast Cancer Screening Program. |
| NBHS_TN* | Nashville Breast Health Study (Triple Negative) | USA | Triple negative invasive breast cancer cases from a collection of Invasive breast cancer or ductal carcinoma in situ cases between the ages of 25 and 75 years (2001-2010). Cases were identified from participating hospitals in the Nashville Metropolitan area and the Tennessee Cancer Registry (TCR). | | Controls were recruited through random digit dialing. |
| OBCS | Oulu Breast Cancer Study[23] | Finland | Consecutive incident cases diagnosed at the Oulu University Hospital between 2000-2004. | | Female blood donors recruited in 2002 from the same geographical region in Northern Finland. |
| OFBCR | Ontario Familial Breast Cancer Registry[24] | Canada | Invasive cases (all aged 20-54 years and a random sample aged 55-69 years) were identified from the Ontario Cancer Registry 1996-1998. All those at high genetic risk (family history of specific cancers particularly breast and ovarian, early onset disease, Ashkenazi ethnicity or a diagnosis of multiple breast cancer) were eligible. Random samples of women not meeting these criteria were also asked to participate. During 2001-2005, some enrollment continued, but was limited to minority and high-risk families. | | Identified by calling randomly selected residential telephone numbers in the same geographical region from 1998-2001; frequency matched to cases by age in 5 year categories. |
| ORIGO | Leiden University Medical Centre Breast Cancer Study[25, 26] | Netherlands | Consecutive case patients diagnosed 1996–2006 in 2 hospitals in South–West Netherlands (Leiden & Rotterdam). No selection for family history; Rotterdam case patients selected for diagnosis aged <70. Case patients with in situ carcinomas eligible. | | (1) Blood bank healthy donors from Southwest Netherlands recruited in 1996, 2000 or 2007; (2) People who married a person who was part of a family with high breast cancer risk (BRCA1/2/x). From the Southwest of the Netherlands, recruited 1990–1996; (3) Females tested at the local clinical genetics department for familial diseases, excluding familial cancer syndromes (no mutation found in gene(s) related to the disease being tested), recruited 1995–2007. |
| OSU* | The Stefanie Spielman Breast Bank and the Columbus Area Control Sample Bank | USA | Incident triple negative breast cancer cases enrolled at The Ohio State University James Comprehensive Cancer Center between 2003 and 2011. | | Controls were chosen from an existing bank of individuals seen for routine health issues at primary care and internal medicine clinics, recruited 2007-2011. They were age and ethnicity frequency matched to cases. |
| PBCS | NCI Polish Breast Cancer Study[27] | Poland | Incident cases identified through a rapid identification system in participating hospitals covering ~ 90% of all eligible cases, and cancer registries in Warsaw and Łódź covering 100% of all eligible cases (2000-2003). | | Randomly selected from population lists of all residents of Poland from 2000-2003, stratified and frequency matched to cases on city and age in 5-year categories. |
| pKARMA | Karolinska Mammography Project for Risk Prediction of Breast Cancer - prevalent cases | Sweden | Incident cases from Jan 2001 – Dec 2008 from the Stockholm/Gotland area. Identified through the Stockholm breast cancer registry. | | Unmatched participants of the KARMA mammography screening study recruited between 2010 and 2011 from Helsingborg and Stockholm. |
| RBCS | Rotterdam Breast Cancer Study[28] | Netherlands | Familial breast cancer patients selected from the clinical genetics center at Erasmus Medical Center between 1994-2005. | | Spouses or mutation-negative siblings of heterozygous Cystic Fibrosis mutation carriers selected from the clinical genetics centre at Erasmus Medical Center between 1996-2006. |
| RPCI* | Roswell Park Cancer Institute | USA | Triple negative invasive breast cancer cases from incident cases recruited to the RPCI Data Bank and Biorepository. | | Healthy controls identified from employee volunteers, and women recruited from community events. |
| SASBAC | Singapore and Sweden Breast Cancer Study[29] | Sweden | Women diagnosed in Sweden aged 50-74 in 1993-1995. | | Population-based controls frequency matched by age to the cases. |
| SBCS | Sheffield Breast Cancer Study[30] | UK | Women with breast cancer recruited in 1998-2005 at surgical outpatient clinics at the Royal Hallamshire Hospital, Sheffield. | | Unselected women attending the Sheffield Mammography Screening Service in 2000-2004 with no evidence of a breast lesion. |
| SEARCH | Study of Epidemiology & Risk Factors in Cancer Heredity[31] | UK | Identified through the Eastern Cancer Registration and Information Centre: (i) prevalent cases; diagnosed 1991-1996; under 55 years of age at diagnosis; recruited 1996-2002 (ii) incidence cases; diagnosed since 1996; under 70 years of age at diagnosis; recruited 1996-present. | | (a) Women from the same geographic region selected from the EPIC-Norfolk cohort study, 1992-1994 (b) women attending GP practices, frequency matched to cases by age and geographic region (2003-present) (c) women attending for breast screening as part of the NHSBSP participating in the Sisters in Breast Screening (SIBS) study |
| SKKDKFZS* | Städtisches Klinikum Karlsruhe Deutsches Krebsforschungszentrum Study[32] | Germany | Triple negative breast cancer cases from the SKKDKFZ breast cancer case cohort study consisting of women diagnosed with primary *in situ* or invasive breast cancer at the Städtisches Klinikum Karlsruhe, Karlsruhe, Germany from 1993 to 2005. Cases were between 21-93 years of age. | | Controls for the triple negative cases selected from GC-HBOC |
| SZBCS | IHCC-Szczecin Breast Cancer Study[33] | Poland | Prospectively ascertained cases of invasive breast cancer patients diagnosed at the Regional Oncology Hospital (2002-2003 and 2006-2007) or the University Hospital (2002-2007), both in Szczecin, West Pomerania, Poland. | | Selected from a population-based study of the 1.3 million inhabitants of West Pomerania (2003-2004); matched to cases for year of birth, sex and region. |
| UKBGS | Breakthrough Generations Study[34] | UK | Cohort members who had had breast cancer or in situ breast cancer before entry into the Breakthrough Generations Study (cohort of >100,000 women followed up for breast cancer, recruited from the UK during 2003-2011). | | Women who had not had breast cancer or in situ breast cancer before entry into the cohort study selected by 1:1 matching to cases on date of birth, year of entry in to the study (2003-2009), source of recruitment, availability of blood sample and ethnicity. |
| **STUDIES OF ASIAN WOMEN** | | | | | |
| ACP | Asian Cancer Project | Thailand | Cases from oncology centres in Thailand that underwent biopsy and had been pathologically diagnosed as having breast cancer. | | Hospital based controls are women who were admitted to the same hospital as the cases with diseases not related to cancer or metabolic syndromes such as diabetes, heart diseases or conditions related to gynaecology. |
| HERPACC | Hospital-based Epidemiologic Research Program at Aichi Cancer Center[35] | Japan | Incident breast cancer cases who first visited Aichi Cancer Center between 2001 and 2005 and were diagnosed within 1 year from the first visit. No previous history of any type of cancer. | | Controls were selected from pool of non-cancer patients who firstly visited Aichi Cancer Center between 2001-2005. Non-cancer status is defined as "having no positive finding on any of clinical/laboratory/graphical examination within 1 year from their fist visit. No previous history of cancer is alllowed. |
| LAABC | Los Angeles County Asian-American Breast Cancer Case-Control Study[36] | USA | Incident cases recruited from 1995-2007 and identified from SEER cancer registries in Los Angeles County. Grouped by self-reported ethnicity. | | Controls were recruited during 1995-2009 and selected from the same neighbourhood as where cancer cases resided at the time of diagnosis. Controls were frequency-matched to the cases on specific Asian ethnicities and 5-year age groups |
| MYBRCA | Malaysian Breast Cancer Genetic Study[37] | Malaysia | Breast cancer cases identified at the Breast Cancer Clinic in University Malaya Medical Centre Jan 2001-July 2010; cases are a mixture of prevalent and incident cases | | Controls are cancer-free individuals (21-70 years) with no breast or ovarian cancer in first-degree relatives, randomly selected from women attending same hospital. |
| SBCGS | Shanghai Breast Cancer Genetic Study[38] | China | Newly diagnosed breast cancer cases recruited from 1996 -2009. Cases were identified mostly from the Shanghai Cancer Registry. Some cases were identified from the Shanghai Women’s Health Study. | | Community controls randomly selected from the general population using the resident registry or from cancer-free cohort members in the Shanghai Women’s Health Study. The controls were recruited from the same geographical region as cases during 1996-2009. |
| SEBCS | Seoul Breast Cancer Study[39] | South Korea | Consecutive, incident, cases from 2 hospitals in Seoul recruited between 2001-2005. | | Women from same catchment area and participating in annual health check-up (2001-2005). |
| SGBCC | Singapore Breast Cancer Cohort | Singapore | Living breast cancer patients diagnosed with primary in-situ or invasive breast cancer at National University Hospital. Cases are a mixture of prevalent and incident cases. | | All community-dwelling individuals who are Singaporeans or Singaporean Permanent Residents, 21 years and older. Exclusion criteria were a medical history of cancer, acute myocardial infarction or stroke, or major psychiatric morbidity including schizophrenia, psychotic depression, and advanced Alzheimer's Disease. |
| TBCS | IARC-Thai Breast Cancer[40] | Thailand | Incident cases diagnosed at the National Cancer Institute (NCI) in Bangkok and Khon Kaen Hospital during 2002-2004. | | Randomly selected women visiting hospital patients with diseases other than breast or ovarian cancer at NCI Bangkok and Khon Kaen Hospital during 2002- 2004). |
| TWBCS | Taiwanese Breast Cancer Study[41] | Taiwan | Incident cases diagnosed & treated at 2 major teaching hospitals in Taiwan between 2002-2005. | | Randomly selected women attending a health examination at same hospitals between 2002-2005. |
| **STUDIES OF AFRICAN-AMERICAN WOMEN** | | | | | |
| NBHS | Nashville Breast Health Study[38] | USA | Through a rapid case-ascertainment system, we identified newly-diagnosed breast cancer cases through the Tennessee State Cancer Registry and five major hospitals in the city that provide medical care for breast cancer patients. Eligible cases were women diagnosed with invasive breast cancer or ductal carcinoma in situ, who were between the ages of 25 and 75, had no prior history of cancer other than non-melanoma skin cancer, had a resident telephone, spoke English, and who were able to provide consent to the study. Recruitment period was from 2001 to 2011. The recruitment for European Americans ended in 2008. | | Controls were identified via random digit dialing (RDD) of households in the same geographic area as cases during 2001-2011. Eligibility criteria for controls were the same as cases with the exception that controls did not have a prior cancer diagnosis other than simple skin cancer. Controls were frequency matched to cases on 5-year age group, race, and county of residence. |
| SCCS | Southern Community Cohort Study[42] | USA | Incident cases (N=222) from 2002-2009 identified from 12 state cancer registries in southeastern US. Prevalent cases (N=493) identified by self-reported history of breast cancer reported on cohort enrollment survey. | | Controls for the incident cases were individually matched on age (+/- 2 years), self-reported race, menopausal status, enrollment site, and date of sample collection (+/- 6 months). Controls for the prevalent cases were frequency matched on age (+/- 1 year) and enrollment site. |

*CTS, NBCS and SKKDKFZ are studies in BCAC but were genotyped as part of the triple negative consortium (TNBCC). Part of GENICA was also genotyped as part of TNBCC. DEMOKRITOS, NBHS_TN, OSU and RPCI were additional non-BCAC studies included as part of the TNBCC.

**Table S1** **references**

1. Dite GS, Jenkins MA, Southey MC, Hocking JS, Giles GG, McCredie MR, Venter DJ, Hopper JL: **Familial risks, early-onset breast cancer, and BRCA1 and BRCA2 germline mutations**. *J Natl Cancer Inst* 2003, **95**(6):448-457.

2. Schmidt MK, Tollenaar RA, de Kemp SR, Broeks A, Cornelisse CJ, Smit VT, Peterse JL, van Leeuwen FE, Van't Veer LJ: **Breast cancer survival and tumor characteristics in premenopausal women carrying the CHEK2*1100delC germline mutation**. *J Clin Oncol* 2007, **25**(1):64-69.

3. Schrauder M, Frank S, Strissel PL, Lux MP, Bani MR, Rauh C, Sieber CC, Heusinger K, Hartmann A, Schulz-Wendtland R *et al*: **Single nucleotide polymorphism D1853N of the ATM gene may alter the risk for breast cancer**. *J Cancer Res Clin Oncol* 2008, **134**(8):873-882.

4. Fletcher O, Johnson N, Palles C, dos Santos Silva I, McCormack V, Whittaker J, Ashworth A, Peto J: **Inconsistent association between the STK15 F31I genetic polymorphism and breast cancer risk**. *J Natl Cancer Inst* 2006, **98**(14):1014-1018.

5. Colleran G, McInerney N, Rowan A, Barclay E, Jones AM, Curran C, Miller N, Kerin M, Tomlinson I, Sawyer E: **The TGFBR1*6A/9A polymorphism is not associated with differential risk of breast cancer**. *Breast Cancer Res Treat* 2010, **119**(2):437-442.

6. Yang R, Dick M, Marme F, Schneeweiss A, Langheinz A, Hemminki K, Sutter C, Bugert P, Wappenschmidt B, Varon R *et al*: **Genetic variants within miR-126 and miR-335 are not associated with breast cancer risk**. *Breast Cancer Res Treat* 2011, **127**(2):549-554.

7. Villeneuve S, Fevotte J, Anger A, Truong T, Lamkarkach F, Gaye O, Kerbrat P, Arveux P, Miglianico L, Imbernon E *et al*: **Breast cancer risk by occupation and industry: analysis of the CECILE study, a population-based case-control study in France**. *Am J Ind Med* 2011, **54**(7):499-509.

8. Weischer M, Bojesen SE, Tybjaerg-Hansen A, Axelsson CK, Nordestgaard BG: **Increased risk of breast cancer associated with CHEK2*1100delC**. *J Clin Oncol* 2007, **25**(1):57-63.

9. Milne RL, Ribas G, Gonzalez-Neira A, Fagerholm R, Salas A, Gonzalez E, Dopazo J, Nevanlinna H, Robledo M, Benitez J: **ERCC4 associated with breast cancer risk: a two-stage case-control study using high-throughput genotyping**. *Cancer Res* 2006, **66**(19):9420-9427.

10. Seal S, Thompson D, Renwick A, Elliott A, Kelly P, Barfoot R, Chagtai T, Jayatilake H, Ahmed M, Spanova K *et al*: **Truncating mutations in the Fanconi anemia J gene BRIP1 are low-penetrance breast cancer susceptibility alleles**. *Nat Genet* 2006, **38**(11):1239-1241.

11. Widschwendter M, Apostolidou S, Raum E, Rothenbacher D, Fiegl H, Menon U, Stegmaier C, Jacobs IJ, Brenner H: **Epigenotyping in peripheral blood cell DNA and breast cancer risk: a proof of principle study**. *PLoS One* 2008, **3**(7):e2656.

12. Pesch B, Ko Y, Brauch H, Hamann U, Harth V, Rabstein S, Pierl C, Fischer HP, Baisch C, Justenhoven C *et al*: **Factors modifying the association between hormone-replacement therapy and breast cancer risk**. *Eur J Epidemiol* 2005, **20**(8):699-711.

13. Justenhoven C, Pierl CB, Haas S, Fischer HP, Baisch C, Hamann U, Harth V, Pesch B, Bruning T, Vollmert C *et al*: **The CYP1B1_1358_GG genotype is associated with estrogen receptor-negative breast cancer**. *Breast Cancer Res Treat* 2008, **111**(1):171-177.

14. Bogdanova N, Cybulski C, Bermisheva M, Datsyuk I, Yamini P, Hillemanns P, Antonenkova NN, Khusnutdinova E, Lubinski J, Dork T: **A nonsense mutation (E1978X) in the ATM gene is associated with breast cancer**. *Breast Cancer Res Treat* 2009, **118**(1):207-211.

15. Hartikainen JM, Tuhkanen H, Kataja V, Dunning AM, Antoniou A, Smith P, Arffman A, Pirskanen M, Easton DF, Eskelinen M *et al*: **An autosome-wide scan for linkage disequilibrium-based association in sporadic breast cancer cases in eastern Finland: three candidate regions found**. *Cancer Epidemiol Biomarkers Prev* 2005, **14**(1):75-80.

16. Beesley J, Jordan SJ, Spurdle AB, Song H, Ramus SJ, Kjaer SK, Hogdall E, DiCioccio RA, McGuire V, Whittemore AS *et al*: **Association between single-nucleotide polymorphisms in hormone metabolism and DNA repair genes and epithelial ovarian cancer: results from two Australian studies and an additional validation set**. *Cancer Epidemiol Biomarkers Prev* 2007, **16**(12):2557-2565.

17. De Maeyer L, Van Limbergen E, De Nys K, Moerman P, Pochet N, Hendrickx W, Wildiers H, Paridaens R, Smeets A, Christiaens M-R *et al*: **Does Estrogen Receptor-Negative/Progesterone Receptor-Positive Breast Carcinoma Exist?** *J Clin Oncol* 2008, **26**(2):335-336.

18. Flesch-Janys D, Slanger T, Mutschelknauss E, Kropp S, Obi N, Vettorazzi E, Braendle W, Bastert G, Hentschel S, Berger J *et al*: **Risk of different histological types of postmenopausal breast cancer by type and regimen of menopausal hormone therapy**. *Int J Cancer* 2008, **123**(4):933-941.

19. Catucci I, Verderio P, Pizzamiglio S, Manoukian S, Peissel B, Barile M, Tizzoni L, Bernard L, Ravagnani F, Galastri L *et al*: **SNPs in ultraconserved elements and familial breast cancer risk**. *Carcinogenesis* 2009, **30**(3):544-545; author reply 546.

20. Olson JE, Ma CX, Pelleymounter LL, Schaid DJ, Pankratz VS, Vierkant RA, Fredericksen ZS, Ingle JN, Wu Y, Couch F *et al*: **A comprehensive examination of CYP19 variation and breast density**. *Cancer Epidemiol Biomarkers Prev* 2007, **16**(3):623-625.

21. Giles GG, English DR: **The Melbourne Collaborative Cohort Study**. *IARC Sci Publ* 2002, **156**:69-70.

22. Nordgard SH, Johansen FE, Alnaes GI, Bucher E, Syvanen AC, Naume B, Borresen-Dale AL, Kristensen VN: **Genome-wide analysis identifies 16q deletion associated with survival, molecular subtypes, mRNA expression, and germline haplotypes in breast cancer patients**. *Genes Chromosomes Cancer* 2008, **47**(8):680-696.

23. Erkko H, Xia B, Nikkila J, Schleutker J, Syrjakoski K, Mannermaa A, Kallioniemi A, Pylkas K, Karppinen SM, Rapakko K *et al*: **A recurrent mutation in PALB2 in Finnish cancer families**. *Nature* 2007, **446**(7133):316-319.

24. John EM, Hopper JL, Beck JC, Knight JA, Neuhausen SL, Senie RT, Ziogas A, Andrulis IL, Anton-Culver H, Boyd N *et al*: **The Breast Cancer Family Registry: an infrastructure for cooperative multinational, interdisciplinary and translational studies of the genetic epidemiology of breast cancer**. *Breast Cancer Res* 2004, **6**(4):R375-389.

25. de Bock GH, Schutte M, Krol-Warmerdam EM, Seynaeve C, Blom J, Brekelmans CT, Meijers-Heijboer H, van Asperen CJ, Cornelisse CJ, Devilee P *et al*: **Tumour characteristics and prognosis of breast cancer patients carrying the germline CHEK2*1100delC variant**. *J Med Genet* 2004, **41**(10):731-735.

26. Huijts PE, Vreeswijk MP, Kroeze-Jansema KH, Jacobi CE, Seynaeve C, Krol-Warmerdam EM, Wijers-Koster PM, Blom JC, Pooley KA, Klijn JG *et al*: **Clinical correlates of low-risk variants in FGFR2, TNRC9, MAP3K1, LSP1 and 8q24 in a Dutch cohort of incident breast cancer cases**. *Breast Cancer Res* 2007, **9**(6):R78.

27. Garcia-Closas M, Brinton LA, Lissowska J, Chatterjee N, Peplonska B, Anderson WF, Szeszenia-Dabrowska N, Bardin-Mikolajczak A, Zatonski W, Blair A *et al*: **Established breast cancer risk factors by clinically important tumour characteristics**. *Br J Cancer* 2006, **95**(1):123-129.

28. Easton DF, Pooley KA, Dunning AM, Pharoah PD, Thompson D, Ballinger DG, Struewing JP, Morrison J, Field H, Luben R *et al*: **Genome-wide association study identifies novel breast cancer susceptibility loci**. *Nature* 2007, **447**(7148):1087-1093.

29. Wedren S, Lovmar L, Humphreys K, Magnusson C, Melhus H, Syvanen AC, Kindmark A, Landegren U, Fermer ML, Stiger F *et al*: **Oestrogen receptor alpha gene haplotype and postmenopausal breast cancer risk: a case control study**. *Breast Cancer Res* 2004, **6**(4):R437-449.

30. MacPherson G, Healey CS, Teare MD, Balasubramanian SP, Reed MW, Pharoah PD, Ponder BA, Meuth M, Bhattacharyya NP, Cox A: **Association of a common variant of the CASP8 gene with reduced risk of breast cancer**. *J Natl Cancer Inst* 2004, **96**(24):1866-1869.

31. Lesueur F, Pharoah PD, Laing S, Ahmed S, Jordan C, Smith PL, Luben R, Wareham NJ, Easton DF, Dunning AM *et al*: **Allelic association of the human homologue of the mouse modifier Ptprj with breast cancer**. *Hum Mol Genet* 2005, **14**(16):2349-2356.

32. Rashid MU, Jakubowska A, Justenhoven C, Harth V, Pesch B, Baisch C, Pierl CB, Bruning T, Ko Y, Benner A *et al*: **German populations with infrequent CHEK2*1100delC and minor associations with early-onset and familial breast cancer**. *Eur J Cancer* 2005, **41**(18):2896-2903.

33. Jakubowska A, Jaworska K, Cybulski C, Janicka A, Szymanska-Pasternak J, Lener M, Narod SA, Lubinski J: **Do BRCA1 modifiers also affect the risk of breast cancer in non-carriers?** *Eur J Cancer* 2009, **45**(5):837-842.

34. Swerdlow AJ, Jones ME, Schoemaker MJ, Hemming J, Thomas D, Williamson J, Ashworth A: **The Breakthrough Generations Study: design of a long-term UK cohort study to investigate breast cancer aetiology**. *Br J Cancer* 2011, **105**(7):911-917.

35. Kawase T, Matsuo K, Suzuki T, Hiraki A, Watanabe M, Iwata H, Tanaka H, Tajima K: **FGFR2 intronic polymorphisms interact with reproductive risk factors of breast cancer: results of a case control study in Japan**. *Int J Cancer* 2009, **125**(8):1946-1952.

36. Wu AH, Yu MC, Tseng CC, Stanczyk FZ, Pike MC: **Dietary patterns and breast cancer risk in Asian American women**. *Am J Clin Nutr* 2009, **89**(4):1145-1154.

37. Thirthagiri E, Lee SY, Kang P, Lee DS, Toh GT, Selamat S, Yoon SY, Taib NA, Thong MK, Yip CH *et al*: **Evaluation of BRCA1 and BRCA2 mutations and risk-prediction models in a typical Asian country (Malaysia) with a relatively low incidence of breast cancer**. *Breast Cancer Res* 2008, **10**(4):R59.

38. Zheng W, Long J, Gao YT, Li C, Zheng Y, Xiang YB, Wen W, Levy S, Deming SL, Haines JL *et al*: **Genome-wide association study identifies a new breast cancer susceptibility locus at 6q25.1**. *Nat Genet* 2009, **41**(3):324-328.

39. Han S, Lee KM, Choi JY, Park SK, Lee JY, Lee JE, Noh DY, Ahn SH, Han W, Kim DH *et al*: **CASP8 polymorphisms, estrogen and progesterone receptor status, and breast cancer risk**. *Breast Cancer Res Treat* 2008, **110**(2):387-393.

40. Sangrajrang S, Schmezer P, Burkholder I, Waas P, Boffetta P, Brennan P, Bartsch H, Wiangnon S, Popanda O: **Polymorphisms in three base excision repair genes and breast cancer risk in Thai women**. *Breast Cancer Res Treat* 2008, **111**(2):279-288.

41. Ding SL, Yu JC, Chen ST, Hsu GC, Kuo SJ, Lin YH, Wu PE, Shen CY: **Genetic variants of BLM interact with RAD51 to increase breast cancer susceptibility**. *Carcinogenesis* 2009, **30**(1):43-49.

42. Signorello LB, Hargreaves MK, Blot WJ: **The Southern Community Cohort Study: investigating health disparities**. *J Health Care Poor Underserved* 2010, **21**(1 Suppl):26-37.
