# Supplementary material for: Genetic variation at CYP3A is associated with age at menarche and breast cancer risk: a case-control study
Source: Breast Cancer Res. 2014 May 26;16(3):R51. doi: 10.1186/bcr3662 (PMC4522594; doi:10.1186/bcr3662)
Supplement: Additional file 1 — Contains Table S1 presenting details of participating BCAC studies; Table S2 presenting rs10235235 genotypes for breast cancer cases and controls from 49 BCAC studies; Table S3 presenting availability of data on age at diagnosis, hormone receptor status, morphology, grade and nodal status for breast cancer cases from 38 European BCAC studies; Table S4 presenting availability of data on age at menarche for breast cancer cases and controls from 40 European BCAC studies; and Figure S1 showing association of the rs10235235-AG genotype with breast cancer risk for women of Asian and African-American ancestry. [file bcr3662-S1.zip › Additional file 1/4703570011029251_add3.docx]

**Figure S1 Association of rs10235235-AG genotype with breast cancer risk for women of Asian and African-American ancestry**

Asian

ACP

HERPACC

LAABC

MYBRCA

SBCGS

SEBCS

SGBCC

TBCS

TWBCS

Subtotal (I-squared = 0.0%, p = 0.706)

African-American

NBHS

SCCS

Subtotal (I-squared = 0.0%, p_het_ = 0.977)

Study

418

560

808

770

829

1114

384

138

774

367

679

Cases

636

1376

990

610

892

1129

502

253

236

252

680

Controls

0.01

0.00

0.00

0.04

0.00

0.00

0.01

0.01

0.00

0.20

0.22

MAF

1.22 (0.58, 2.55)

1.24 (0.23, 6.80)

8.90 (1.04, 76.30)

0.81 (0.49, 1.33)

1.09 (0.27, 4.39)

1.10 (0.40, 3.02)

1.52 (0.50, 4.66)

1.06 (0.76, 1.49)

1.09 (0.77, 1.54)

1.10 (0.87, 1.38)

1.09 (0.90, 1.32)

OR (95% CI)

1.22 (0.58, 2.55)

1.24 (0.23, 6.80)

8.90 (1.04, 76.30)

0.81 (0.49, 1.33)

1.09 (0.27, 4.39)

1.10 (0.40, 3.02)

1.52 (0.50, 4.66)

1.06 (0.76, 1.49)

1.09 (0.77, 1.54)

1.10 (0.87, 1.38)

1.09 (0.90, 1.32)

OR (95% CI)

1

.5

1

2

Forest plot of the association of rs10235235-AG (heterozygote) genotype with breast cancer risk for women of Asian and African-American ancestry. MAF, minor allele frequency. Horizontal lines represent 95% CIs. Square boxes represent study specific fixed-effects estimates. Diamonds represent the combined, fixed-effects estimate of the OR and 95% CI for Asian and African-American studies. The vertical line represents the null effect (OR=1.0). rs10235235 is rare in Asian populations (MAF=0.002). The heterozygote OR could not be estimated in two studies SEBCS (one carrier among 1,114 cases and no carriers among 1,129 controls) and TWBCS (no carriers among 774 cases and one carrier among 236 controls)(see Additional file 1: Table S2). A Forest plot of homozygote ORs is not presented for non-European studies due to the rarity of rs10235235 in Asian populations and the small number of African-American studies (N=2).
